# Association between the presence of delirium during intensive care unit admission and cognitive impairment or psychiatric problems: the Korean ICU National Data Study

**DOI:** 10.1186/s40560-022-00598-4

**Published:** 2022-02-14

**Authors:** Ryoung-Eun Ko, Danbee Kang, Hyejung Park, Juhee Cho, Gee Young Suh, Chi Ryang Chung

**Affiliations:** 1 Department of Critical Care Medicine, Samsung Medical Center, Sungkyunkwan University School of Medicine, 81 Irwon-Ro, Gangnam-gu, Seoul, 06351 Republic of Korea; 2 Center for Clinical Epidemiology, Samsung Medical Center, Seoul, Republic of Korea; 3 Department of Clinical Research Design & Evaluation, SAIHST, Sungkyunkwan University, Seoul, Republic of Korea; 4 Division of Pulmonary and Critical Care Medicine, Department of Medicine, Samsung Medical Center, Sungkyunkwan University, Seoul, Republic of Korea; 5 Department of Medicine, Samsung Medical Center, Sungkyunkwan University, Seoul, Republic of Korea

**Keywords:** Cognition, Delirium, Intensive care unit, Nationwide cohort study, Psychiatric

## Abstract

**Objective:**

Delirium in the intensive care unit (ICU) may be a preventable risk factor for cognitive impairment or psychiatric problems. We aimed to evaluate the association between the presence of delirium during hospitalization involving ICU care and post-discharge cognitive impairment or psychiatric problems.

**Design:**

A retrospective cohort study.

**Setting:**

A database of nationwide insurance claims data.

**Patients:**

All adult patients aged 18 years or older who were admitted to an ICU between January 1, 2008, and May 31, 2015, and had no history of previous cognitive impairment or psychiatric problems were included in the study.

**Interventions:**

None.

**Measurements and main results:**

Of 306,011 patients who met the inclusion criteria, the proportion of those who experienced delirium during hospitalization was 55.0% (*n* = 168,190). The patients with delirium during hospitalization had significantly increased odds for cognitive impairment (adjusted hazard ratio [HR] 1.17; 95% confidence interval [CI] 1.05–1.29) and psychiatric problems (adjusted HR 1.78; 95% CI 1.67–1.90) after discharge compared with patients without delirium. In patients who had delirium, the incidence of cognitive impairment was 210.8 per 1000 person-years. In 19,496 patients who were diagnosed with cognitive impairment, depression (*n* = 3233, 16.5%), sleep disorder (*n* = 1791, 9.2%), and anxiety (*n* = 1683, 8.6%) were commonly co-diagnosed. The most common psychiatric problem was sleep disorder (148.7 per 1000 person-years), followed by depression (133.3 per 1000 person-years).

**Conclusions:**

Among patients received ICU care, those who experienced delirium during hospitalization had an increased risk of developing cognitive impairment or psychiatric problems post-discharge. Many patients showed multiple cognitive impairment and psychiatric problems during the follow-up period. Efforts to decrease these problems should be made to increase the quality of life of these ICU survivors.

**Supplementary Information:**

The online version contains supplementary material available at 10.1186/s40560-022-00598-4.

## Background

The number of patients admitted to the intensive care unit (ICU) is increasing, and more than 80% of patients survive ICU care [1, 2]. Accordingly, increasing attention is being given to the long-term outcome of ICU survivors. Previous studies have shown that ICU survivors are affected by a wide range of physical, cognitive, and psychiatric issues [3, 4]. These phenomena, also referred to as “post-intensive care syndrome (PICS)”, decrease the patients’ quality of life and deteriorate their ability to return to the workforce [1, 5]. Thus, the Society of Critical Care Medicine released the ABCDEF bundle, which encourages assessment and management of pain, agitation, and delirium, implementation of early mobilization, and family engagement, along with a recommended strategy to mitigate the risk of PICS [6, 7].

Cognitive impairment and psychiatric problems worsen the quality of life of patients who have recovered from a critical illness, and they have become important public health issues [8–11]. Hopkins and colleagues reported that 45% of ICU survivors had cognitive sequelae and 29% had symptoms of depression and anxiety at 1 year [9]. Sivanathan and colleagues found that ICU patients had a higher rate of diagnoses related to new psychiatric problems in the year after discharge than other hospitalized patients [11]. Therefore, various efforts, including early mobilization, minimization of sedation, and emotional and psychological support, have been made to prevent cognitive impairment and psychiatric problems in patients receiving ICU care [12].

Delirium is a frequent disorder encountered in ICU patients and is associated with substantially increased morbidity and mortality after ICU care [13–15]. Despite various efforts, a recent meta-analysis reported that the prevalence of delirium in ICU patients remains high, at approximately 31% [16]. Several studies have demonstrated the impact of delirium on cognitive impairment and psychiatric problems [4, 17–20]. However, these previous studies were small prospective cohort studies and limited to showing the impact of delirium on detailed post-ICU problems and medical costs. Therefore, we conducted this nationwide cohort study to evaluate the association between the presence of delirium during hospitalization involving ICU care and post-discharge cognitive impairment or psychiatric problems.

## Methods

### Study population

**Fig. 1:**
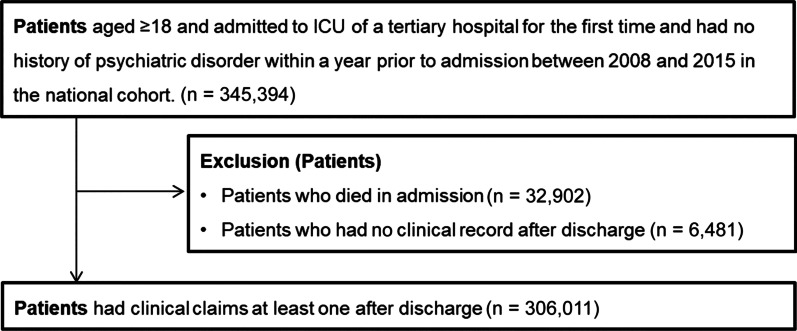
Flow diagram

We analyzed a retrospective cohort from the Korean ICU National Data (KIND) Study [21] based on the Health Insurance Review and Assessment (HIRA) database from the Korean Ministry of Health. The KIND database include all ICU admissions in Korea. From the KIND database, we selected initial ICU admissions in adults (aged over 18 years) without a history of clinic visits for psychiatric disorders and psychiatric medications at admission between January 1, 2007, and May 31, 2016 (n = 345,394). Among the participants, we excluded patients who died before discharge (*n* = 32,902) and those who had no clinical records after discharge (*n* = 6481). The final sample size was 306,011 (198,491 men and 107,520 women) (Fig. 1).

The study was reviewed by the Institutional Review Board (IRB) of Samsung Medical Center (IRB protocol 2015-11-17) and was exempted from the informed consent requirement, because we used only de-identified previously collected administrative data.

### Data collection and clinical outcomes

Delirium was defined as the presence of psychiatric disease codes (F-code) or a code for antipsychotic or anxiolytic medications in a prescription claim within an admission. We also conducted a sensitivity analysis that used a more specific definition of delirium: the presence of the code for delirium (F05.x except F05.1) instead of all psychiatric disease codes. Comorbidities were defined as a 1-year history of any disease before admission to the ICU and summarized using the Charlson’s comorbidity index [22].

The primary outcome was cognitive impairment and psychiatric problems within 2 years of discharge. Cognitive impairment was defined as the presence of dementia (F00, F01, F03, F03, G30, and G31) or cognitive dysfunction (R41) codes or prescriptions of anti-dementia agents such as cholinesterase inhibitors (donepezil, rivastigmine, and galantamine), memantine, choline alfoscerate, oxiracetam, nicergoline, acetyl l-carnitine, and piracetam for at least 28 days. Psychological impairment was defined as the presence of disease codes for depression (F32, F33, and F34.1), anxiety (F40 and F41), somatoform/conversion disorder (F43), substance abuse disorder (F10–F19), and sleep disorder (F51 and G47) or prescription of antidepressants or antipsychotic agents for at least 28 days. The secondary outcomes were readmissions to the hospital, readmissions to the ICU, and medical costs during hospitalization and after discharge.

Data on underlying diseases and comorbidities, procedures, prescriptions, and patient demographics were obtained from claims data and defined using International Classification of Diseases 10th revision codes [23]. Comorbidities were defined as a 1-year history of any disease before admission to the ICU [24, 25].

Admissions were classified as medical or surgical. Medical admissions were defined as admissions to one of the following departments: pediatrics, internal medicine, neurology, neuropsychiatry, dermatology, rehabilitation medicine, general medicine, radiology, family medicine, and emergency medicine. Surgical admissions were defined as admissions to one of the following departments: general surgery, orthopedic surgery, neurosurgery, thoracic and cardiovascular surgery, plastic surgery, ophthalmology, otorhinolaryngology, urology, oral surgery, anesthesiology, and obstetrics and gynecology.

The quality of ICU care was determined using the codes of nurse staffing grades (AJ11–AJ19, AJ21–AJ9, and AJ31–AJ39). Nurse staffing grades (ratio of number of beds to number of nurses) were categorized into nine grades (grades 1–9). Ratios of number of beds to number of nurses of < 0.5 and ≥ 2.5 indicated grade 1 (best) and grade 9 (worst), respectively.

The procedures of interest were mechanical ventilation (Korean National Health Insurance procedure codes: M5857, M5858, and M5860), extracorporeal membrane oxygenation (ECMO; Korean National Health Insurance procedure codes: O1901–O1904), and continuous renal replacement therapy (CRRT; Korean National Health Insurance procedure codes: O7031–O7034 and O7051–O7054). The use of vasopressor drugs such as dobutamine, dopamine, epinephrine, and norepinephrine for > 2 days was determined according to the Korean Drug and Anatomical Therapeutic Chemical Codes (148201BIJ, 38900BIJ, 148701BIJ, 148702BIJ, 429500BIJ, 152601BIJ, and 203101BIJ) [25]. Advanced interventions were defined as procedures, such as mechanical ventilation, ECMO, CRRT, and administration of vasopressor drugs.

In-hospital cost was defined as the sum of the insurance payment costs incurred during hospital admission, and post-ICU cost was defined as the sum of payments per visit during the 2 years after discharge. To obtain the cost related to cognitive impairment or psychiatric problems, we summed all costs related to codes with cognitive impairment or psychiatric problems as the principal reason during 2 years after discharge.

### Statistical analyses

Data are presented as means with standard deviations or medians and interquartile ranges for continuous variables, and as numbers and percentages for categorical variables. Demographic characteristics were compared between the delirium and non-delirium groups using the Mann–Whitney *U* test for continuous variables and Pearson’s Chi-square test for categorical variables. Patients were followed up until the development of a study endpoint, readmission, 2 years after discharge, or available last clinic visit upto the end of the study period (May 31, 2016). Cumulative incidences and hazard rates were estimated using the Kaplan–Meier method. We calculated the hazard ratios (HRs) with 95% confidence intervals (CIs) for developing each clinical outcome using a proportional hazards regression model. For the cost analysis, we used log-transformed cost as the outcome (because the costs were markedly right-skewed) and estimated the ratio of cost (with 95% CI). Post ICU cost were obtained by the incremental healthcare costs after discharging ICU. With consideration the different follow-up period for each patient, we calculated the PPPM (per patient per month) cost. Each patient’s total cost during the follow-up was divided by the corresponding patient’s follow-up period. All analyses were adjusted for potential confounding factors, such as age, sex, comorbidities, medical beneficiaries, region, mechanical ventilation, ECMO, CRRT, vasopressor drugs, quality of ICU, and length of ICU stay more than 7 days. Statistical significance was set at *p* < 0.05. Statistical analyses were performed using SAS^®^ Visual Analytics (SAS Institute Inc., USA).

## Results

Of the all 306,011 patients, the proportion of those with delirium was 55.0% (*n* = 168,190). There were 64.9% men, and the mean patient age was 58.3 (15.1) years (Table 1). Patients with delirium were more likely to undergo advanced interventions during hospital admission and showed increased length of ICU stay (3 [1–6] versus 2 [0–4] days, *p* < 0.001) and hospital day (16 [9–30] versus 8 [4–14] days, *p* < 0.001) (Table 1). Moreover, patients with delirium were more likely to be admitted to the medical department, than those without delirium, although the reasons for admission were not significantly different between the two groups (Additional file 1: Table S1). The Charlson’s comorbidity index was higher in patients with delirium than in those without delirium (2 [0–4] versus 2 [0–3], *p* < 0.001). Furthermore, congestive heart failure (8.3% versus 7.1%, *p* < 0.001), chronic pulmonary disease (26.5% versus 24.9%, *p* < 0.001), connective tissue disease (3.1% versus 2.9%, *p* = 0.002), liver disease (23.6% versus 22.4%, *p* < 0.001), paraplegia and hemiplegia (1.3% versus 1.1%, *p* < 0.001), and cancer (21.0% versus 19.8%, *p* < 0.001) were more common in patients with delirium than in those without delirium.

**Table 1:** Characteristics of study participants

Variables	Patients with delirium (*n* = 168,190)	Patients without delirium (*n* = 137,821)	*p*
Age, years	57.9 (12.3)	58.7 (15.0)	< 0.001
Sex, male	108,766 (64.7)	89,725 (65.1)	0.01
Admitted department			< 0.001
Medical	95,570 (56.8)	60,504 (43.9)	
Surgical	72,620 (43.2)	77,317 (56.1)	
Charlson’s comorbidity index	2 (0–4)	2 (0–3)	< 0.001
Comorbidities			
Myocardial infarction	5709 (3.4)	5593 (4.1)	< 0.001
Congestive heart failure	14,017 (8.3)	9809 (7.1)	< 0.001
Peripheral vascular disease	19,178 (11.4)	15,511 (11.3)	0.2
Cerebrovascular disease	21,771 (12.9)	18,805 (13.6)	< 0.001
Chronic pulmonary disease	44,498 (26.5)	34,262 (24.9)	< 0.001
Connective tissue disease	5150 (3.1)	3959 (2.9)	0.002
Peptic ulcer disease	40,118 (23.9)	32,633 (23.7)	0.26
Liver disease	39,718 (23.6)	30,881 (22.4)	< 0.001
Diabetes mellitus	44,871 (26.7)	37,531 (27.2)	< 0.001
Paraplegia and hemiplegia	2252 (1.3)	1449 (1.1)	< 0.001
Renal disease	7039 (4.2)	5667 (4.1)	0.31
Cancer	35,351 (21.0)	27,319 (19.8)	< 0.001
AIDS/HIV	83 (0.0)	58 (0.0)	0.35
Medical beneficiaries	5030 (3.0)	3443 (2.5)	< 0.001
Region			< 0.001
Seoul	86,242 (51.3)	62,832 (45.6)	
Metropolitan area	44,620 (26.5)	42,065 (30.5)	
Rural area	37,328 (22.2)	32,924 (23.9)	
Advanced intervention			
Mechanical ventilation	62,242 (37.0)	6166 (4.5)	< 0.001
ECMO	903 (0.5)	34 (0.0)	< 0.001
Hemodialysis	4464 (2.7)	868 (0.6)	< 0.001
Vasopressor drugs	84,240 (50.1)	33,118 (24)	< 0.001
ICU length of stay, days	3 (1–6)	2 (0–4)	< 0.001
ICU admission over 7 days	41,786 (24.8)	8406 (6.1)	< 0.001
Hospital length of stay, days	16 (9–30)	8 (4–14)	< 0.001
Quality of ICU			< 0.001
Grade 1	49,633 (29.5)	37,495 (27.2)	
Grade 2	38,051 (22.6)	30,708 (22.3)	
Grade 3	55,629 (33.1)	53,005 (38.5)	
Grade ≥ 4	24,877 (14.8)	16,613 (12.1)	

Data are presented number (percentage), mean (standard deviation) or median (interquartile range)

*AIDS* acquired immune deficiency syndrome, *HIV* human immunodeficiency virus, *ECMO* extracorporeal membrane oxygenation, *ICU* intensive care unit

**Fig. 2:**
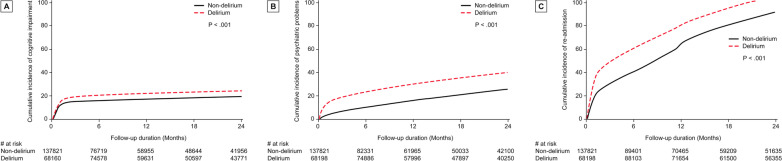
Cumulative incidences of outcomes in patients with and without delirium. **a** Cognitive impairment, **b** psychiatric problems, and **c** readmission

The incidence rates of cognitive impairment and psychiatric problems were higher in patients with delirium than in those without delirium (Table 2). The HR for cognitive impairment and psychiatric problems was 1.25 (95% CI 1.07–1.48) and 1.80 (95% CI 1.67–1.94) in patients with delirium compared with those without delirium, respectively. After adjusting for potential confounding factors, the HRs for cognitive impairment and psychiatric problems were also significant at 1.17 (95% CI 1.05–1.29) and 1.78 (95% CI 1.67–1.90) in patients with delirium compared with those without delirium, respectively. In the sensitivity analysis, which used a specific definition of delirium, the HRs for cognitive impairment and psychiatric problems were 1.45 (95% CI 1.40–1.51) and 1.40 (95% CI 1.31–1.51) in patients with delirium compared with those without delirium, respectively (Additional file 1: Table S2). After adjusting for confounding factors, the adjusted HR for cognitive impairment remained significant (1.10; 95% CI 1.07–1.13). In patients who needed advanced interventions, the HR for cognitive impairment was not different (1.08; 95% CI 0.83–1.40, *p* = 0.59), whereas the HR for psychiatric impairment presented a significant difference (1.70; 95% CI 1.59–1.82) (Table 2). The cumulative incidences of cognitive impairment, psychiatric problems, and hospital readmission were significantly higher in patients with delirium than compared in those without delirium (Fig. 2).

**Table 2:** Hazard ratio (95% CI) for cognitive impairment and psychiatric problems within 2 years of discharge in patients with delirium versus patients without delirium

	Delirium		Non-delirium		Univariable		Multivariable^a^	
	Number of cases	Incidence rate, (1000 person-years)	Number of cases	Incidence rate, (1000 person-years)	HR (95% CI)	*p*	HR (95% CI)	*p*
Overall (*n* = 306,011)								
Cognitive impairment	27,462	210.8	20,017	156.2	1.25 (1.07–1.48)	0.006	1.17 (1.05–1.29)	0.004
Psychiatric problems	37,574	293.4	20,736	153.4	1.80 (1.67–1.94)	< 0.001	1.78 (1.67–1.90)	< 0.001
Readmission to hospital	107,207	695.7	81,300	539.4	1.22 (1.20–1.24)	< 0.001	1.24 (1.21–1.26)	< 0.001
Readmission to ICU	23,685	86.6	16,891	71.5	1.16 (1.14–1.19)	< 0.001	1.12 (1.10–1.14)	< 0.001
Patients who needed advanced interventions (*n* = 137,833)								
Cognitive impairment	14,813	197.0	5592	179.7	1.08 (0.83–1.40)	0.59	1.08 (0.91–1.29)	0.39
Psychiatric problems	20,983	286.3	5414	161.3	1.70 (1.59–1.82)	< 0.001	1.63 (1.55–1.72)	< 0.001
Readmission to hospital	64,056	735.3	23,370	624.5	1.15 (1.09–1.21)	< 0.001	1.07 (1.03–1.10)	< 0.001
Readmission to ICU	14,909	94.5	5012	80.5	1.17 (1.09–1.24)	< 0.001	1.03 (0.98–1.09)	0.23

*CI* confidence interval, *HR* hazard ratio, *ICU* intensive care unit

^a^Adjusted for age, sex, comorbidities, medical beneficiaries, region, mechanical ventilation, ECMO, hemodialysis, vasopressor drugs, quality of ICU, and ICU length of stay more than 7 days

In patients with delirium, the most common psychiatric impairment was sleep disorder (148.7/1000 person-years), followed by depression (133.3/1000 person-years) and anxiety (123.5/1000 person-years, Additional file 1: Table S3). In patients without delirium, the most common psychiatric impairment was anxiety (86.1/1000 person-years), followed by sleep disorder (77.9/1000 person-years) and depression (60.2/1000 person-years). After adjusting for potential confounding factors, the HRs for sleep disorder, depression, anxiety, substance abuse disorder, and stress reaction/conversion disorder were significantly increased in patients with delirium compared with those without delirium.

**Fig. 3:**
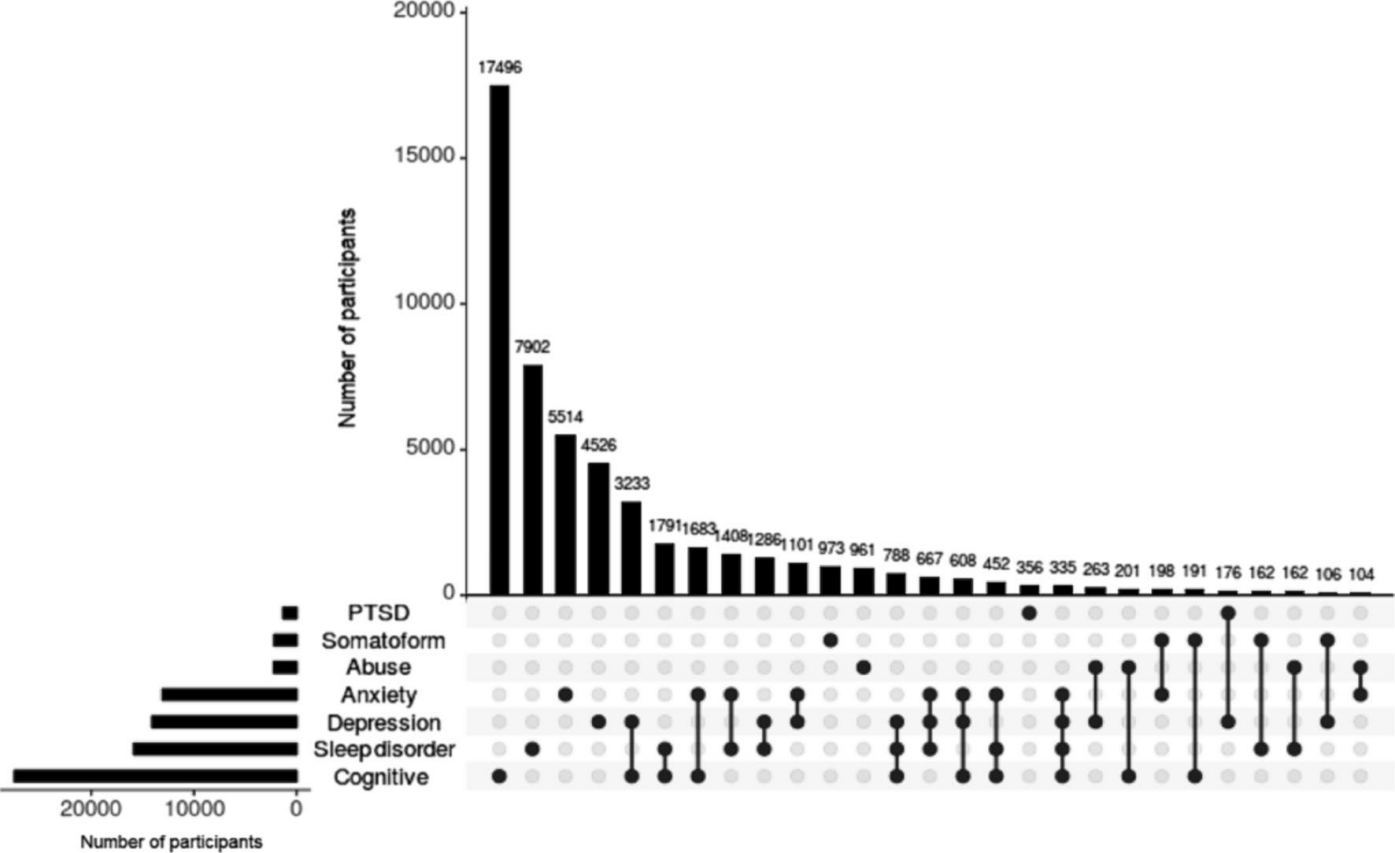
UpSet plot of the combinations of cognitive impairment and detailed psychiatric problems. The vertical bars reflect the number of participants who experienced a specific combination of cognitive impairment and detailed psychiatric problems, identified by dots below each bar. The horizontal bars reflect the number of participants who experienced each cognitive impairment and detailed psychiatric problems across all combinations of cognitive impairment and detailed psychiatric problems

Most common disorder across all combinations of cognitive impairment and detailed psychiatric problems was cognitive impairment alone, followed by sleep disorder alone and anxiety alone. Cognitive impairment and depression frequently co-occurred (*n* = 3233). Cognitive impairment also co-occurred with sleep disorder (*n* = 1791) and anxiety (*n* = 1683) (Fig. 3).

With respect to cost outcomes, an average of $7693 was spent in the ICU during hospitalization. The cost was $9429 and $5732 in patients with and without delirium, respectively. Patients with delirium spent 1.26 times (95% CI 1.26–1.27) more than patients without delirium. In terms of post-ICU cost, the average cost was $5268. Patients with and without delirium spent $6377 and $5082, respectively, and patients with delirium spent 1.15 times (95% CI 1.14–1.17) more than patients without delirium. Among the post-ICU expenses, the cost related to cognitive impairment or psychiatric problems related was $141 and $75 in patients with and without delirium, respectively. Patients with delirium also had higher cost related to cognitive and psychiatric impairments after ICU admission than those without delirium (Table 3). When we compare to the PPPM as consider the follow-up duration, the results were also similar with total cost (Table 3).

**Table 3:** Ratio (95% CI) of medical costs in patients with delirium compared with patients without delirium

	Patients with delirium (*n* = 168,190)	Patients without delirium (*n* = 137,821)	Adjusted^a^ ratio (95% CI)	*p*
In-hospital cost, $	9429 (5843–15,856)	5732 (3381–8155)	1.26 (1.26–1.27)	< 0.001
Post-ICU cost, $ (PPPM)				
Total	139.5 (54.6–640.8)	106.0 (51.7–297.1)	1.16 (1.14–1.17)	< 0.001
Cognitive impairment or psychiatric problems related	3.2 (0.8–17.8)	2.0 (0.6–9.7)	1.45 (1.37–1.53)	< 0.001

Data are presented median (interquartile range)

*CI* confidence interval, *ICU* intensive care unit, *PPPM* per-patient-per-months

1 US dollar = 1022 Korean won (exchange rate as of May 31, 2014)

^a^Adjusted for age, sex, comorbidities, medical beneficiaries, region, mechanical ventilation, ECMO, CRRT, vasopressor drugs, quality of ICU, and ICU length of stay more than 7 days

## Discussion

In this study, we investigated the association between the presence of delirium during hospitalization involving ICU care and post-discharge cognitive impairment or psychiatric problems. The key strength of this study is the first national cohort study to show the association between delirium and cognitive impairment or psychiatric problems seen in a small prospective cohort from previous studies. In addition, this study included a long post-discharge period and demonstrated the impact of delirium on post-ICU medical costs.

### Delirium increases the risk of cognitive impairment

In this nationwide cohort study, we found that the incidence of cognitive impairment was 15.5% within 2 years after hospital discharge in ICU survivors. Similar to previous reports, this study showed that patients with delirium had an increased risk of cognitive impairment compared with those without delirium (HR, 1.17; 95% CI 1.05–1.29). Previous studies have shown that delirium is a significant independent risk factor for long-term cognitive impairment [4, 26–28]. Pandharipande and colleagues conducted a prospective observational study to identify potentially modifiable risk factors of long-term cognitive impairment in mixed ICU patients. They observed that a longer duration of delirium during hospitalization was associated with worse cognition at 3 and 12 months [4]. Girard and colleagues also found that delirium was associated with long-term cognitive impairment according to several clinical phenotypes (i.e., hypoxic, septic, sedative-associated, metabolic, and unclassified) at 3 and 12 months of follow-up. Their study showed that sedation, hypoxia, and sepsis-related delirium increased the risk of long-term cognitive impairment [27]. Considering that cognitive impairment is a common and severe complication and affects the long-term quality of life of ICU survivors [4, 12], efforts to prevent delirium may be warranted to reduce the risk of long-term cognitive impairment.

### Delirium increases the risk of psychiatric disorder

In previous studies, comorbid conditions, benzodiazepine prescription, and preexisting psychiatric disorders were associated with an increased risk of psychiatric problems experienced by ICU survivors [29, 30]. The association between delirium and psychiatric problems is controversial. Jacson and colleagues conducted a prospective multicenter cohort study with 821 patients and reported that delirium was not consistently associated with psychiatric problems [2]. In contrast, some studies have shown that delirium is associated with psychiatric problems [31–33]. In the current study, we found that the presence of delirium during hospitalization involving ICU care increased the risk of psychiatric problems within 2 years of discharge (HR, 1.78; 95% CI 1.67–1.90). In detail, the risk of substance abuse disorder, sleep disorder, or depression was significantly increased in patients who experienced delirium compared with those who did not. We analyzed patients who were hospitalized with ICU admission, and the results suggested that delirium in the ICU and immediately after the ICU stay may be important with respect to long-term psychiatric problems. Further studies are needed to determine the relationship between the timing of delirium and the development of psychiatric problems.

### Association between cognitive impairment and psychiatric problems

Physical problems, cognitive impairment, and psychiatric problems might be distinct domains of critical illness rather than a component of a single unifying syndrome [34, 35]. In addition, various types of psychiatric problems in ICU survivors may present as sleep disorder, depression, anxiety, or substance abuse disorder. In the current study, we evaluated the number of patients who experienced cognitive impairment or various types of psychiatric problems and documented cognitive impairments that often manifest alongside depression, sleep disorder, or anxiety. Similar to our results, previous studies have reported that depression, anxiety, and post-traumatic stress disorder are associated with worsening cognitive function [36–38]. Bruck and colleagues reported a correlation between self-rated cognitive function and psychological symptoms [36]. Mikkelsome and colleagues reported that cognitive dysfunction was often associated with increased anxiety in long-term survivors of acute respiratory distress syndrome who received ICU care [38]. Importantly, it was found that one ICU survivor often experienced various psychiatric problems within 2 years. Therefore, monitoring for detailed cognitive impairment or psychiatric problems is necessary to provide appropriate management for PICS.

### Delirium increases medical costs

The in-hospital and post-ICU costs were higher in patients who experienced delirium than in those who did not. In addition, the risk of increased costs related to cognitive impairment or psychiatric problems after discharge was significantly higher in patients with delirium than in those without delirium (HR, 1.50; 95% CI 1.44–1.56). Previous studies have identified a link between delirium and in-hospital cost. Milbrandt and colleagues conducted prospective cohort study of mechanical ventilated patients, and revealed that delirium is associated with significantly higher intensive care unit and hospital costs [39]. Similar results were seen in the post-hoc analysis of the BRAIN-ICU cohort [40]. This study found that cost of delirium increased after adjusting for time-varying severity of illness and length of stay. Increased medical costs may be linked to the characteristics of patients who experienced delirium during hospitalization involving ICU care. These patients may have increased comorbidities, a need for advanced interventions, and increased ICU and hospital lengths of stay. One interesting finding in this study is that delirium affect post-ICU costs. This results might be correlated with increased hospital readmissions and higher post-ICU medical costs [41, 42]. These results suggest that efforts to prevent delirium should be made to reduce the overall ICU-related medical costs.

### Limitations

Several limitations of this study need to be acknowledged. First, owing to the use of retrospective national registry data, detailed clinical data, including the reasons for ICU admission and laboratory tests, could not be collected. Second, the HIRA does not provide any survival information after hospital discharge; thus, long-term mortality outcomes could not be evaluated. Third, there might be difference in severity between the two groups, which may have influenced the results. To minimize the risk of severity imbalance, we adjusted mechanical ventilation, ECMO, hemodialysis, and vasopressor drugs. Fourth, since delirium and cognitive impairment were identified based on diagnostic codes with medication, the incidence could underestimation regarding the mild cases. Therefore, delirium was defined as the presence of psychiatric disease codes or a code for antipsychotic or anxiolytic medications. In addition, a sensitivity analysis was performed in the presence of the code for delirium. Fifth, we did not have information on cognitive function at admission, which could be potential confounders for increased cognitive impairment risk after discharge. However, we tried to exclude severe cognitive impairment at admission as including only patient without a history of any psychiatric clinic visit or psychiatric medication. Finally, since data on clinical outcomes was based on claims data, there might be misclassification of cognitive impairment and hard to know the exact date at onset of cognitive impairment. However, the NHIS routinely audits the claims, and the data for clinical outcomes are considered highly reliable and have been used in numerous peer-reviewed publications.

## Conclusion

In summary, this study evaluated the impact of delirium on cognitive impairment and psychiatric problems. In patients who received ICU care, those who had delirium during hospitalization had an increased risk of experiencing cognitive impairment or psychiatric problems after discharge. Many patients showed multiple cognitive impairments and psychiatric problems during the follow-up period. The in-hospital and post-ICU costs were higher in patients who experienced delirium than in those who did not.

## Supplementary Information

**Additional file 1: Table S1.** Cause of admission. **Table S2.** Hazard ratio (95% CI) for cognitive impairment or psychiatric problems within 2 years of discharge in patients with delirium versus patients without delirium using a conservative definition of delirium. **Table S3.** Hazard ratio (95% CI) for psychiatric problems within 2 years of discharge in patients with delirium versus patients without delirium

## Data Availability

The data that support the findings of this study are available on request from the corresponding author. The data are not publicly available due to privacy or ethical restrictions.

## Abbreviations

CRRT: Continuous renal replacement therapy
ECMO: Extracorporeal membrane oxygenation
HIRA: Health Insurance Review and Assessment
ICU: Intensive care unit
PICS: Post-intensive care syndrome

## References

[1] Needham DM, Davidson J, Cohen H, Hopkins RO, Weinert C, Wunsch H (2012). Improving long-term outcomes after discharge from intensive care unit: report from a stakeholders' conference. Crit Care Med.

[2] Jackson JC, Pandharipande PP, Girard TD, Brummel NE, Thompson JL, Hughes CG (2014). Depression, post-traumatic stress disorder, and functional disability in survivors of critical illness in the BRAIN-ICU study: a longitudinal cohort study. Lancet Respir Med.

[3] Parker AM, Sricharoenchai T, Raparla S, Schneck KW, Bienvenu OJ, Needham DM (2015). Posttraumatic stress disorder in critical illness survivors: a metaanalysis. Crit Care Med.

[4] Pandharipande PP, Girard TD, Jackson JC, Morandi A, Thompson JL, Pun BT (2013). Long-term cognitive impairment after critical illness. N Engl J Med.

[5] Hashem MD, Nallagangula A, Nalamalapu S, Nunna K, Nausran U, Robinson KA (2016). Patient outcomes after critical illness: a systematic review of qualitative studies following hospital discharge. Crit Care.

[6] Barr J, Fraser GL, Puntillo K, Ely EW, Gelinas C, Dasta JF (2013). Clinical practice guidelines for the management of pain, agitation, and delirium in adult patients in the intensive care unit. Crit Care Med.

[7] Devlin JW, Skrobik Y, Gelinas C, Needham DM, Slooter AJC, Pandharipande PP (2018). Clinical Practice Guidelines for the Prevention and Management of Pain, agitation/sedation, delirium, immobility, and sleep disruption in adult patients in the ICU. Crit Care Med.

[8] Rothenhausler HB, Ehrentraut S, Stoll C, Schelling G, Kapfhammer HP (2001). The relationship between cognitive performance and employment and health status in long-term survivors of the acute respiratory distress syndrome: results of an exploratory study. Gen Hosp Psychiatry.

[9] Hopkins RO, Weaver LK, Chan KJ, Orme JF (2004). Quality of life, emotional, and cognitive function following acute respiratory distress syndrome. J Int Neuropsychol Soc.

[10] Kapfhammer HP, Rothenhausler HB, Krauseneck T, Stoll C, Schelling G (2004). Posttraumatic stress disorder and health-related quality of life in long-term survivors of acute respiratory distress syndrome. Am J Psychiatry.

[11] Sivanathan L, Wunsch H, Vigod S, Hill A, Pinto R, Scales DC (2019). Mental illness after admission to an intensive care unit. Intensive Care Med.

[12] Karnatovskaia LV, Johnson MM, Benzo RP, Gajic O (2015). The spectrum of psychocognitive morbidity in the critically ill: a review of the literature and call for improvement. J Crit Care.

[13] Pisani MA, Kong SY, Kasl SV, Murphy TE, Araujo KL, Van Ness PH (2009). Days of delirium are associated with 1-year mortality in an older intensive care unit population. Am J Respir Crit Care Med.

[14] van den Boogaard M, Schoonhoven L, Evers AW, van der Hoeven JG, van Achterberg T, Pickkers P (2012). Delirium in critically ill patients: impact on long-term health-related quality of life and cognitive functioning. Crit Care Med.

[15] Salluh JI, Wang H, Schneider EB, Nagaraja N, Yenokyan G, Damluji A (2015). Outcome of delirium in critically ill patients: systematic review and meta-analysis. BMJ.

[16] Krewulak KD, Stelfox HT, Leigh JP, Ely EW, Fiest KM (2018). Incidence and prevalence of delirium subtypes in an adult ICU: a systematic review and meta-analysis. Crit Care Med.

[17] Girard TD, Jackson JC, Pandharipande PP, Pun BT, Thompson JL, Shintani AK (2010). Delirium as a predictor of long-term cognitive impairment in survivors of critical illness. Crit Care Med.

[18] Wolters AE, Peelen LM, Welling MC, Kok L, de Lange DW, Cremer OL (2016). Long-term mental health problems after delirium in the ICU. Crit Care Med.

[19] Grover S, Sahoo S, Chakrabarti S, Avasthi A (2019). Post-traumatic stress disorder (PTSD) related symptoms following an experience of delirium. J Psychosom Res.

[20] Lin SM, Liu CY, Wang CH, Lin HC, Huang CD, Huang PY (2004). The impact of delirium on the survival of mechanically ventilated patients. Crit Care Med.

[21] Park J, Jeon K, Chung CR, Yang JH, Cho YH, Cho J (2018). A nationwide analysis of intensive care unit admissions, 2009–2014—The Korean ICU National Data (KIND) study. J Crit Care.

[22] Charlson ME, Pompei P, Ales KL, MacKenzie CR (1987). A new method of classifying prognostic comorbidity in longitudinal studies: development and validation. J Chronic Dis.

[23] Lee YS, Lee YR, Chae Y, Park SY, Oh IH, Jang BH (2016). Translation of Korean medicine use to ICD-codes using National Health Insurance Service-National Sample Cohort. Evid Based Complement Alternat Med.

[24] Kim KH (2010). Comparative study on three algorithms of the ICD-10 Charlson comorbidity index with myocardial infarction patients. J Prev Med Public Health.

[25] Korea Pharmaceutical Information Service. Korea Pharmaceutical Information. https://www.kpis.or.kr. Accessed 1 June 2021.

[26] Witlox J, Eurelings LS, de Jonghe JF, Kalisvaart KJ, Eikelenboom P, van Gool WA (2010). Delirium in elderly patients and the risk of postdischarge mortality, institutionalization, and dementia: a meta-analysis. JAMA.

[27] Girard TD, Thompson JL, Pandharipande PP, Brummel NE, Jackson JC, Patel MB (2018). Clinical phenotypes of delirium during critical illness and severity of subsequent long-term cognitive impairment: a prospective cohort study. Lancet Respir Med.

[28] Gunther ML, Morandi A, Krauskopf E, Pandharipande P, Girard TD, Jackson JC (2012). The association between brain volumes, delirium duration, and cognitive outcomes in intensive care unit survivors: the VISIONS cohort magnetic resonance imaging study*. Crit Care Med.

[29] Schandl A, Bottai M, Hellgren E, Sundin O, Sackey PV (2013). Developing an early screening instrument for predicting psychological morbidity after critical illness. Crit Care.

[30] Wade DM, Howell DC, Weinman JA, Hardy RJ, Mythen MG, Brewin CR (2012). Investigating risk factors for psychological morbidity three months after intensive care: a prospective cohort study. Crit Care.

[31] Dolan MM, Hawkes WG, Zimmerman SI, Morrison RS, Gruber-Baldini AL, Hebel JR (2000). Delirium on hospital admission in aged hip fracture patients: prediction of mortality and 2-year functional outcomes. J Gerontol A Biol Sci Med Sci.

[32] Fann JR, Alfano CM, Roth-Roemer S, Katon WJ, Syrjala KL (2007). Impact of delirium on cognition, distress, and health-related quality of life after hematopoietic stem-cell transplantation. J Clin Oncol.

[33] Rothenhausler HB, Grieser B, Nollert G, Reichart B, Schelling G, Kapfhammer HP (2005). Psychiatric and psychosocial outcome of cardiac surgery with cardiopulmonary bypass: a prospective 12-month follow-up study. Gen Hosp Psychiatry.

[34] Marra A, Pandharipande PP, Girard TD, Patel MB, Hughes CG, Jackson JC (2018). Co-occurrence of post-intensive care syndrome problems among 406 survivors of critical illness. Crit Care Med.

[35] Maley JH, Brewster I, Mayoral I, Siruckova R, Adams S, McGraw KA (2016). Resilience in survivors of critical illness in the context of the survivors’ experience and recovery. Ann Am Thorac Soc.

[36] Bruck E, Schandl A, Bottai M, Sackey P (2018). The impact of sepsis, delirium, and psychological distress on self-rated cognitive function in ICU survivors-a prospective cohort study. J Intensive Care.

[37] Duggan MC, Wang L, Wilson JE, Dittus RS, Ely EW, Jackson JC (2017). The relationship between executive dysfunction, depression, and mental health-related quality of life in survivors of critical illness: results from the BRAIN-ICU investigation. J Crit Care.

[38] Mikkelsen ME, Shull WH, Biester RC, Taichman DB, Lynch S, Demissie E (2009). Cognitive, mood and quality of life impairments in a select population of ARDS survivors. Respirology.

[39] Milbrandt EB, Deppen S, Harrison PL, Shintani AK, Speroff T, Stiles RA (2004). Costs associated with delirium in mechanically ventilated patients. Crit Care Med.

[40] Vasilevskis EE, Chandrasekhar R, Holtze CH, Graves J, Speroff T, Girard TD (2018). The cost of ICU delirium and coma in the intensive care unit patient. Med Care.

[41] Prescott HC, Langa KM, Liu V, Escobar GJ, Iwashyna TJ (2014). Increased 1-healthcare use in survivors of severe sepsis. Am J Respir Crit Care Med.

[42] Prescott HC, Langa KM, Iwashyna TJ (2015). Readmission diagnoses after hospitalization for severe sepsis and other acute medical conditions. JAMA.

