# Supplementary material for: Association between the presence of delirium during intensive care unit admission and cognitive impairment or psychiatric problems: the Korean ICU National Data Study
Source: J Intensive Care. 2022 Feb 14;10:7. doi: 10.1186/s40560-022-00598-4 (PMC8842893; doi:10.1186/s40560-022-00598-4)
Supplement: Supplementary file 1 — Additional file 1: Table S1. Cause of admission. Table S2. Hazard ratio (95% CI) for cognitive impairment or psychiatric problems within 2 years of discharge in patients with delirium versus patients without delirium using a conservative definition of delirium. Table S3. Hazard ratio (95% CI) for psychiatric problems within 2 years of discharge in patients with delirium versus patients without delirium [file 40560_2022_598_MOESM1_ESM.docx]

***Table S1:* Cause of admission**

|  | **Total**  **(N=306,011)** | **Patients who had delirium (n=168,190)** | **Patients without delirium**  **(n=137,821)** |
| --- | --- | --- | --- |
| Diseases of the circulatory system (I00-I99) | 147,127 (48·1) | 73,878 (43·9) | 73,249 (53·1) |
| Neoplasms (C00-D48) | 68,238 (22·3) | 40,178 (23·9) | 28,060 (20·4) |
| Injury, poisoning and certain other consequences of external causes (S00-T98) | 24,805 (8·1) | 14,982 (8·9) | 9,823 (7·1) |
| Diseases of the digestive system (K00-K93) | 17,516 (5·7) | 9,175 (5·5) | 8,341 (6·1) |
| Diseases of the respiratory system (J00-J99) | 9,571 (3·1) | 6,391 (3·8) | 3,180 (2·3) |
| Diseases of the nervous system (G00-G99) | 7,069 (2·3) | 4,381 (2·6) | 2,688 (2·0) |
| Diseases of the genitourinary system (N00-N99) | 6,681 (2·2) | 3,495 (2·1) | 3,186 (2·3) |
| Certain infectious and parasitic diseases (A00-B99) | 5,353 (1·7) | 3,373 (2·0) | 1,980 (1·4) |
| Diseases of the musculo-skeletal system and connective tissue (M00-M99) | 5,168 (1·7) | 3,188 (1·9) | 1,980 (1·4) |
| Congenital malformations, deformations and chromosomal abnormalities (Q00-Q99) | 3,620 (1·2) | 2,811 (1·7) | 809 (0·6) |
| Endocrine, nutritional and metabolic disease (E00-E90) | 2,464 (0·8) | 1,288 (0·8) | 1,176 (0·9) |
| Symptoms, signs and abnormal clinical and laboratory findings, not elsewhere classified (R00-R99) | 2,333 (0·8) | 1,214 (0·7) | 1,119 (0·8) |
| Factors influencing health status and contact with health services (Z00-Z99) | 2,309 (0·8) | 1,482 (0·9) | 827 (0·6) |
| Pregnancy, childbirth and the puerperium (O00-O99) | 1,959 (0·6) | 1,078 (0·6) | 881 (0·6) |
| Mental and behavioral disorders (F00-F99) | 614 (0·2) | 614 (0·4) | 0 (0·0) |
| Diseases of the blood and blood -forming organs and certain disorders involving the immune mechanism (D50-D89) | 540 (0·2) | 277 (0·2) | 263 (0·2) |
| Diseases of the skin and subcutaneous tissue (L00-L99) | 357 (0·1) | 215 (0·1) | 142 (0·1) |
| Diseases of the eye and adnexa (H00-H59) | 103 (0·0) | 54 (0·0) | 49 (0·0) |
| Diseases of the ear and mastoid process (H60-H95) | 93 (0·0) | 53 (0·0) | 40 (0·0) |

***Table S2:* Hazard ratio (95% CI) for cognitive impairment or psychiatric problems within 2 years of discharge in patients with delirium versus patients without delirium using a conservative definition of delirium**^*^

|  | **Delirium** | | **Non-delirium** | | **Univariable** | | **Multivariable^**^** | |
| --- | --- | --- | --- | --- | --- | --- | --- | --- |
|  | **Number of cases** | **Incidence rate,**  **(1,000 person–years)** | **Number of cases** | **Incidence rate,**  **(1,000 person–years)** | **HR (95% CI)** | **p** | **HR (95% CI)** | **p** |
| Cognitive impairment | 16,641 | 945·1 | 171,866 | 598·3 | 1·45 (1·40–1·51) | <0·001 | 1·10 (1·07–1·13) | <0·001 |
| Psychiatric impairment | 3,983 | 111·0 | 36,593 | 77·2 | 1·40 (1·31–1·51) | <0·001 | 1·04 (0·99–1·10) | 0·09 |
| Readmission to hospital | 8,570 | 327·3 | 60,685 | 147·2 | 1·91 (1·70–2·15) | <0·001 | 1·42 (1·32–1·53) | <0·001 |
| Readmission to ICU | 11,260 | 474·4 | 87,447 | 218·9 | 1·99 (1·88–2·10) | <0·001 | 1·66 (1·57–1·75) | <0·001 |

HR=hazard ratio. CI=confidence interval. ICU=intensive care unit.

^*^ Delirium was defined as the presence of delirium codes and use of antipsychotic or anxiolytic medications during admission.

^**^Adjusted for age, sex, comorbidities, medical beneficiaries, region, mechanical ventilation, ECMO, hemodialysis, vasopressor drugs, quality of ICU, and ICU length of stay more than 7 days.

***Table S3:* Hazard ratio (95% CI) for psychiatric problems within 2 years of discharge in patients with delirium versus patients without delirium**

|  | **Delirium** | | **Non-delirium** | | **Univariable** | | **Multivariable^*^** | |
| --- | --- | --- | --- | --- | --- | --- | --- | --- |
|  | **Number of cases** | **Incidence rate,**  **(1,000 person–years)** | **Number of cases** | **Incidence rate,**  **(1,000 person–years)** | **HR (95% CI)** | **p** | **HR (95% CI)** | **p** |
| Psychiatric problems |  |  |  |  |  |  |  |  |
| Sleep disorder | 15,839 | 148·7 | 8,145 | 77·9 | 1·85 (1·73–1·97) | <0·001 | 1·78 (1·68–1·90) | <0·001 |
| Depression | 14,072 | 133·3 | 6,292 | 60·2 | 2·12 (1·92–2·35) | <0·001 | 2·04 (1·88–2·23) | <0·001 |
| Anxiety | 13,051 | 123·5 | 8,902 | 86·1 | 1·41 (1·32–1·51) | <0·001 | 1·50 (1·41–1·58) | <0·001 |
| Substance abuse disorder | 2,215 | 20·0 | 569 | 5·3 | 3·07 (3·18–4·30) | <0·001 | 4·38 (3·68–5·22) | <0·001 |
| Somatoform/conversion disorder | 2,173 | 19·7 | 2,157 | 20·4 | 0·97 (0·89–1·05) | 0·41 | 1·04 (0·96–1·13) | 0·32 |
| Stress reaction/conversion disorder | 1,296 | 11·7 | 618 | 5·8 | 2·00 (1·78–2·25) | <0·001 | 1·91 (1·68–2·18) | <0·001 |

CI=confidence interval. HR=hazard ratio.

^*^Adjusted for age, sex, comorbidities, medical beneficiaries, region, mechanical ventilation, ECMO, hemodialysis, vasopressor drugs, quality of ICU, and ICU length of stay more than 7 days.
